# Subcutaneous fatty acid composition of steers finished as weanlings or yearlings with and without growth promotants

**DOI:** 10.1186/2049-1891-4-41

**Published:** 2013-11-04

**Authors:** Cletos Mapiye, Tyler D Turner, John A Basarab, Vern S Baron, Jennifer L Aalhus, Michael ER Dugan

**Affiliations:** 1 Agriculture and Agri-Food Canada, Lacombe Research Centre, 6000 C & E Trail, Lacombe, Alberta T4L 1 W1, Canada; 2 Alberta Agriculture and Rural Development, Lacombe Research Centre, 6000 C & E Trail, Lacombe, Alberta T4L 1 W1, Canada

**Keywords:** Age at feedlot entry, Beef, Fatty acids, Growth implant, Steers

## Abstract

**Background:**

The current study evaluated the subcutaneous fatty acid (FA) composition of calf- and yearling-fed steers with or without growth promoting implants. Crossbred steers (n = 112; 267 ± 5.0 kg) of the same contemporary group were allocated to one of four production system and implant strategy based treatments in a completely randomized design with a 2 × 2 factorial arrangement of treatments.

**Results:**

There were no interactions (*P* > 0.05) between production systems and growth promoting implants for the total and individual subcutaneous FA. Yearling as opposed to calf finishing reduced (*P* < 0.05) subcutaneous proportions of C20:3*n*-6, *trans (t)*12-18:1, C14:0, several minor *cis*-monounsaturated FA (*c*-MUFA; *c*9-14:1, *c*11-16:1, *c*11-18:1, *c*12-18:1, *c*13-18:1, *c*9-20:1 and *c*11-20:1), and increased (*P* < 0 .05) subcutaneous proportions of *t*11*c*15-18:2, total and individual branched-chain FA. Subcutaneous fat from steers implanted with growth promotants had higher (*P* < 0.05) proportions of total polyunsaturated FA (PUFA), total *n*-6 PUFA, C18:2*n*-6 and individual *t-*18:1 isomers (*t*6 to *t*10) compared to non-implanted steers.

**Conclusions:**

Overall, current findings show that production systems and growth promotants led to only minor differences in subcutaneous FA composition of beef steers.

## Background

In North America, beef cattle are typically finished using calf or yearling production systems. In calf production systems, cattle enter the feedlot immediately after weaning at 6–8 mo of age and are fed a high-energy finishing diet *ad libitum* until slaughter at 12–14 mo of age to take advantage of the faster growth of large-framed cattle [1,2]. On the contrary, in yearling production systems cattle graze pasture and/or crop residues post-weaning prior to entering a feedlot at 15–18 mo of age to allow for growth of frame in small- to medium-framed cattle and takes advantage of lower priced forages and subsequent compensatory growth in the feedlot. Although age at feedlot entry has been reported to consistently affect fat deposition [3,4], little is known about its effect on fatty acid (FA) composition. It has been suggested that cattle entering a feedlot as yearlings may have a healthier FA profile from a consumer’s perspective due to forage-based diets compared to those entering the feedlot as weanlings [5,6].

Growth promoting implants characterised as being estrogenic (*e.g.,* estradiol benzoate and estradiol) or androgenic (*e.g.,* trenbolone acetate and progesterone) are used extensively in calf and yearling production systems to increase growth rates, carcass yield [3,7] and to reduce carcass fatness [8,9]. Regarding FA composition, studies with estrogen- or androgen-implanted steers showed small increases in the proportions of saturated FA (SFA) and decreases in proportions of monounsaturated FA (MUFA) [10,11]. In contrast, other studies with estrogen-implanted bull calves [12] or steers [13] found reduced SFA and increased MUFA. In the same studies, Hozler et al. [12] and Ibrahim et al. [13] reported a decrease and an increase in the proportions of polyunsaturated FA (PUFA), respectively. In general, the available information on the effects of growth promoting implants on beef FA composition are inconclusive. In addition, the interactive effects of implant strategies with age entering the feedlot have not been investigated. The objective of the present study was, therefore, to determine the FA composition of subcutaneous adipose tissue from beef steers finished as weanlings or yearlings with and without growth promotants. More specifically the objectives were to determine the extent reduced adiposity can influence concentrations of rumen derived FA such as conjugated linoleic acid (CLA), *trans* (*t*)-18:1 isomers and branched-chain FA (BCFA). This report is part of a detailed study which also investigated greenhouse gas emissions [14], economic profitability [15], carcass merit traits [4], beef quality attributes [16] and beef texture [17].

## Materials and methods

### Animal management and treatments

Cattle used in this study were cared for under the guidelines provided by the Canadian Council on Animal Care [18] and the experimental procedures were approved by the Lacombe Research Centre Animal Care Committee. One hundred and twelve crossbred Hereford-Aberdeen Angus (n = 64) or Charolais-Red Angus (n = 48) steers born in March and April 2008 at the Lacombe Research Centre were used in the current study. The management of the cow-calf herd has been previously described by Basarab et al. [19]. Calves were weaned at an average age of 182 d. At weaning, calves were assigned to one of four production system and implant strategy based treatments in a completely randomized design (CRD) with a 2 × 2 factorial arrangement of treatments. There were four pens per treatment (seven steers per pen). Each breed cross was equally represented across treatments.

Post-weaning management of calf- and yearling-finished steers were detailed by López-Campos et al. [15]. In summary, following weaning, calf-finished steers (n = 56; 268 ± 5.4 kg; 191 ± 3 d) were adjusted from a high forage-based diet to a high-grain finishing diet over 42 d and subsequently finished on a high-grain diet containing 81.4% barley grain-based concentrate, 8.9% barley silage and 7.9% grass silage on DM basis for 86 d. After weaning, 56 steer calves (266 ± 4.6 kg, 193 ± 3 d), assigned to the yearling production system, rotationally grazed alfalfa (*Medicago sativa* L.)/meadow brome grass (*Bromus riparius* Rehm.) pasture (fall pasture) for 52 d. Thereafter, a grower diet (on DM basis) containing 43.1% barley silage, 41.1% grass hay and 15.8% rolled barley:oat (60:40) grain mix was fed for 192 d prior to grazing alfalfa/ meadow brome grass pasture (summer pasture) for 90 d. Yearling steers were then placed into a feedlot pen and allowed 21–23 d to adapt to the high-grain diet before finished on a high-grain diet (on DM basis) comprised of 79.0% barley grain-based concentrate and 21.0% barley silage for a period of 86 d. Half of the calf-finished steers (n = 28) were implanted with 20 mg estradiol benzoate and 200 mg progesterone (Synovex-S) at weaning and 120 mg trenbolone acetate and 24 mg estradiol (Revalor-S) 90 d before slaughter. Yearling-finished steers (n = 28) were implanted with Synovex-S at weaning and re-implanted with Synovex-S 83 d after weaning (second time), 71 d after the second implant, 86 d after the third implant and finally implanted with Revalor-S 90 d before slaughter.

### Feed analyses

Feed samples for the fall pasture were collected twice, initially when the cattle went onto pasture and then when the cattle came off pasture. Feed samples for the summer grazing period (June to August 2009) were collected twice per mo, once early and once late, from each of three paddocks where the animals were grazing in a particular month. Finishing feed samples of the total mixed ration for the steers were collected weekly, pooled monthly and analyzed for nutrient and fatty acid composition. Feed analysis procedures of the experimental finishing diets fed to weanling and yearling steers are detailed by López-Campos et al. [15] and Girard et al. [16]. Fatty acid methyl esters (FAME) from the finishing total mixed ration were prepared as described by Sukhija and Palmquist [20] and analyzed using the chromatographic conditions reported Dugan et al. [21].

### Animal slaughter and sample collection

For both production systems, weanling and yearling steers were targeted to be harvested in four groups of 14 animals at a constant backfat end point of 8–10 mm between the 12^th^ and 13^th^ rib over the right *longissimus thoracis* muscle of each animal which corresponded to 11–14 and 19–23 mo of age, respectively. Backfat thickness was measured on the first and last day of feed intake by a certified ultrasound technician using an Aloka 500 V diagnostic real-time ultrasound with a 17 cm 3.5 Mhz linear array transducer (Overseas Monitor Corporation Ltd., Richmond, B.C., Canada) following procedures of Brethour [22]. At one to two wk intervals steers were trucked 3 km for slaughter at the Lacombe Research Centre abattoir such that there were seven implanted and seven non-implanted steers within each slaughter group. At slaughter, final live weights were recorded and animals were stunned, exsanguinated and dressed in a commercial manner. At approximately, 20 min *post-mortem*, during evisceration, a cube of subcutaneous fat (5 cm × 5 cm × the thickness of subcutaneous fat) was collected from the posterior end of the 12^th^ rib and stored at -80°C for subsequent FA analysis.

### Subcutaneous fatty acid analysis

Subcutaneous fat samples (50 mg) were freeze-dried and directly methylated with sodium methoxide according to Cruz-Hernandez et al. [23]. Internal standard (1 mL of 1 mg 23:0 methyl ester/ml toluene; EMD Chemicals Inc. Darmstadt, Germany) was added before the addition of the methylating reagent. The majority of FAME were analysed with gas chromatography (GC) using a 175°C temperature program as described by Dugan et al. [21].

For the identification of FAME by GC, the reference standard no. 601 from Nu-Check Prep Inc, Elysian, MN, USA was used. Branched-chain FAME were identified using a GC reference standard BC-Mix1 purchased previously from Applied Science (State College, PA, USA). *Trans*-18:1 isomers and other PUFA biohydrogenation intermediates, not included in the standard mixtures, were identified by their retention times and elution order [21,23,24]. The FAME were quantified using chromatographic peak area and internal standard (23:0 methyl esters)-based calculations (mg FAME = FAME peak area × relative response factor × mg internal standard added / internal standard peak area). The FAME concentrations were reported as percentage of total FA identified.

### Statistical analysis

Fatty acid data were analyzed using PROC MIXED procedures [25] as a CRD with a 2 × 2 factorial arrangement of treatments. The model fitted production system (calf-fed, yearling-fed), implant strategy (implant, no implant) and their interaction as the main effects and the random effects of pen nested within production system × implant strategy interaction. Initial body weight was included as a covariate. Treatment means were determined using the LSMEANS and PDIFF options and separated using the LSD test. Significance was declared at *P* < 0.05.

## Results and discussion

### Nutritional composition of the experimental diets and steers subcutaneous fat thickness

Overall, the nutritional composition of the finisher diets were similar (Table 1), except that calcium was slighlty higher for the yearling-fed steers compared to calf- fed steers. Total FA content and FA composition of the finishing diets were similar (Table 1) with the diet fed to yearling-finished steers having slightly more linoleic acid (C18:2*n*-6, LA) than that fed to calf-finished steers. Alpha-linolenic acid (C18:3*n*-3, ALA) was the dominant FA in fall and summer pasture grazed by yearling-finished steers (Table 1). End ultrasound subcutaneous thickness of yearling-fed steers (10.1 ± 0.30 mm) was slightly higher than that of calf-fed steers (8.4 ± 0.30 mm).

**Table 1:** Nutritional and fatty acid composition (% of total fatty acids) of the diets fed to calf- and yearling-finished steers

	**Calf-fed steers**	**Yearling-fed steers**			
**Variable**	**Finishing diet**	**Fall pasture**	**Grower diet**	**Summer pasture**	**Finishing diet**
*Nutrient, % DM*					
Crude protein	13.2 ± 1.09	9.40 ± 2.83	12.6 ± 1.52	13.4 ± 2.51	12.6 ± 0.49
ADF	15.2 ± 3.68	36.8 ± 4.54	35.3 ± 2.32	34.4 ± 2.71	16.7 ± 1.22
NDF	27.6 ± 4.45	55.6 ± 3.86	55.1 ± 1.81	56.7 ± 2.68	28.1 ± 1.88
Calcium	0.6 ± 0.19	0.7 ± 0.13	0.7 ± 0.07	0.8 ± 0.21	0.9 ± 0.27
Phosphorus	0.4 ± 0.09	0.3 ± 0.03	0.3 ± 0.03	0.3 ± 0.14	0.5 ± 0.06
TDN, %	75.2 ± 1.72^x^	57.0 ± 5.91^y^	59.0 ± 1.08^y^	60.2 ± 3.53^y^	74.5 ± 0.57^z^
ME^2^, MJ/kg DM	11.4 ± 0.26^x^	8.61 ± 0.89 ^y^	8.91 ± 0.16^y^	9.1 ± 0.53^y^	12.6 ± 0.49^z^
Fatty acid, % total fatty acids					
C16:0	18.7 ± 1.40	24.0 ±1.32	22.1 ± 0.71	28.0 ± 3.82	18.8 ± 0.41
C18:0	2.15 ± 0.17	2.51 ± 0.06	2.07 ± 0.22	2.73 ± 0.56	1.75 ± 0.09
*c*9-18:1	20.8 ± 1.88	9.67 ± 6.32	14.1 ± 5.37	4.67 ± 2.12	18.5 ±0.27
*c*11-18:1	4.37 ± 0.61	2.23 ± 0.60	2.62 ± 0.86	1.39 ± 0.54	4.00 ± 0.26
C18:2*n*-6	43.3 ± 0.87	23.7 ± 8.40	28.8 ± 2.39	15.2 ± 0.21	46.5 ± 0.71
C18:3*n*-3	9.20 ± 0.72	30.1 ± 10.3	26.1 ± 8.09	38.7 ± 8.69	9.00 ± 0.56
Total fatty acids (mg/g DM)	2.30 ± 0.69	1.00 ± 0.69	1.25 ± 0.69	0.96 ± 0.69	2.34 ± 0.69

^x^Equations used to calculate total digestible nutrients (TDN) and metabolizable energy (ME) for the finishing diet fed to calf-fed steers: %TDN = 82.299 - (ADF, % × 0.467); ME, MJ/kg DM = ((%TDN/100) × 4.4 × 0.82) x 4.184 MJ/Mcal); ^y^Equations used to calculate TDN and ME for the pasture, grower’s diet and summer pasture fed to yearling steers: %TDN = 82.299 - (ADF, % × 0.467); ME, MJ/kg DM = ((%TDN/100) × 4.4 × 0.82) × 4.184 MJ/Mcal); ^z^Equations used to calculate TDN and ME for the finishing diet fed to yearling steers: %TDN = 82.299 - (ADF, % × 0.467); ME, MJ/kg DM = ((%TDN/100) × 4.4 × 0.82) x 4.184 MJ/Mcal).

### Effects of production system on fatty acid composition

There were no significant (*P* > 0.05) interactions between production system and growth implant strategy for total and individual FA in subcutaneous fat, and therefore only main effects were reported. Subcutaneous fat from yearling steers had greater (*P* > 0.05) total FA content than calf-finished steers (Table 2) and may relate to the maturity of the depot [26]. Subcutaneous fat from calf- and yearling-finished steers had similar (*P* > 0.05) proportions of total PUFA. The proportions of total and individual *n*-6 PUFA were not affected by production system except for C20:3*n-*6 which was slightly higher (*P* < 0.05) in calf-finished steers than in yearling-finished steers (Table 2). The effect of production systems on C20:3*n*-6 were, however, small and would be of limited biological importance. Alpha-linolenic acid, the only *n-*3 PUFA identified, was not influenced (*P* > 0.05) by production system (Table 2).

**Table 2:** Effect of production system and growth implant strategy on fatty acid composition (% of total fatty acids) from subcutaneous fat of feedlot steers

	**Production system**		**Implant strategy**			***P*-value**	
**Variable**	**Calf-finished**	**Yearling-finished**	**No**	**Yes**	**s.e.m**	**PS**	**IS**
Total fatty acids (g/g of tissue)	0.90^b^	0.93^a^	0.92	0.90	0.01	0.01	0.91
∑ PUFA	1.62	1.69	1.55^b^	1.77^a^	0.07	0.54	0.01
∑ *n*-6	1.39	1.42	1.30^b^	1.51^a^	0.06	0.74	<0.001
C18:2*n*-6	1.32	1.36	1.24^b^	1.45^a^	0.06	0.67	<0.001
C20:2*n*-6	0.02	0.02	0.02	0.02	0.002	0.71	0.49
C20:3*n*-6	0.05^a^	0.04^b^	0.05	0.04	0.004	0.05	0.89
*n*-3							
C18:3*n*-3	0.23	0.27	0.25	0.26	0.01	0.09	0.26
∑ AD	0.61	0.64	0.62	0.63	0.02	0.35	0.71
*c*9,*t*13-/*t*8,*c*13-18:2	0.20	0.22	0.21	0.20	0.01	0.28	0.31
*t*8,*c*12-/*c*9,*t*12-18:2	0.11	0.12	0.11	0.12	0.01	0.37	0.11
*t*9,*c*12-18:2	0.07^a^	0.02^b^	0.05	0.05	0.01	<0.001	0.95
*t*11,*c*15-18:2	0.04^b^	0.12^a^	0.07	0.09	0.01	<0.001	0.09
*c*9,*c*15-18:2	0.19	0.18	0.19	0.18	0.01	0.56	0.35
∑ CLA	0.54	0.49	0.51	0.52	0.02	0.14	0.63
*t*7,*c*9-/*c*9,*t*11-18:2	0.48	0.41	0.44	0.45	0.02	0.12	0.76
*c*10,*t*12-18:2	0.02	0.02	0.02	0.02	0.01	0.11	0.73
*t*9,*t*11-18:2	0.04	0.05	0.04	0.05	0.01	0.21	0.35
∑ *t*18:1	3.29	3.19	3.00	3.46	0.36	0.49	0.61
*t*6/*t*7/*t*8-18:1	0.25	0.25	0.23^b^	0.28^a^	0.01	0.98	<0.001
*t*9-18:1	0.27	0.27	0.26^b^	0.28^a^	0.01	0.72	0.01
*t*10-18:1	1.57	1.65	1.43^b^	1.79^a^	0.12	0.67	0.01
*t*11-18:1	0.67	0.54	0.60	0.61	0.06	0.23	0.92
*t*12-18:1	0.14^a^	0.10^b^	0.12	0.12	0.01	0.01	0.46
*t*13/*t*14-18:1	0.27	0.27	0.26	0.27	0.01	0.70	0.29
*t*16/*c*14-18:1	0.12	0.10	0.11	0.11	0.01	0.31	0.93
∑ MUFA	51.4	51.9	52.1	51.2	0.52	0.63	0.14
∑ *c*-MUFA	50.0	50.0	11.2	10.4	0.05	0.50	0.85
*c*9-14:1	1.70^a^	1.32^b^	1.59^a^	1.43^b^	0.06	<0.001	0.05
*c*7-16:1	0.15	0.15	0.15	0.15	0.01	0.54	0.45
*c*9-16:1	5.24	4.71	5.16	4.80	0.17	0.09	0.15
*c*11-16:1	0.37^a^	0.29^b^	0.35	0.32	0.02	<0.001	0.07
*c*9-17:1	0.51^b^	1.31^a^	0.91	0.910	0.13	0.02	0.99
*c*9-18:1	39.0	39.5	39.6	38.9	0.39	0.43	0.16
*c*11-18:1	1.79	1.76	1.84	1.71	0.06	0.79	0.13
*c*12-18:1	0.10^a^	0.08^b^	0.09	0.09	0.01	0.03	0.34
*c*13-18:1	0.68^a^	0.58^b^	0.66	0.60	0.03	0.04	0.15
*c*16-18:1	0.06	0.05	0.05	0.05	0.01	0.26	0.68
*c*9-20:1	0.09^a^	0.02^b^	0.06	0.05	0.01	0.01	0.18
*c*11-20:1	0.32^a^	0.26^b^	0.31^a^	0.27^b^	0.01	0.02	0.01
∑ BCFA	1.14^b^	1.45^a^	1.28	1.32	0.01	0.05	0.96
*iso-*15:0	0.11^b^	0.16^a^	0.13	0.13	0.01	0.01	0.84
*anteiso*-15:0	0.15^b^	0.19^a^	0.17	0.18	0.01	<0.001	0.19
*iso-*16:0	0.14^b^	0.22^a^	0.18	0.18	0.01	<0.001	0.83
*iso-*17:0	0.32^b^	0.35^a^	0.33	0.34	0.01	0.05	0.27
a*nteiso*-17:0	0.56^b^	0.66^a^	0.59^b^	0.63^a^	0.01	<0.001	0.01
*iso-*18:0	0.12^b^	0.17^a^	0.15	0.14	0.01	<0.001	0.46
∑ SFA	42.2	41.9	41.7	42.4	0.52	0.75	0.23
C14:0	3.85^a^	3.39^b^	3.65	3.57	0.08	0.01	0.43
C15:0	0.47^b^	0.65^a^	0.56	0.56	0.01	<0.001	0.94
C16:0	27.5	26.7	27.2	26.9	0.31	0.13	0.54
C17:0	1.02	1.09	0.99	1.12	0.14	0.81	0.11
C18:0	9.34	10.1	9.23	10.2	0.35	0.20	0.09
C19:0	0.25	0.24	0.24	0.25	0.01	0.65	0.38
C20:0	0.04	0.04	0.04	0.05	0.01	0.84	0.73

^a,b,c^Means with different superscripts for a particular FA profile are significantly different (*P* < 0.05); s.e.m, standard error of mean; PS, production system effect; IS, Implant strategy effect; *c*, *cis*; *t, trans*; ∑ PUFA, sum of polyunsaturated FA = C18:3*n-*3 + C18:2*n-*6 + C20:2*n-*6 + C20:3*n*-6; ∑ *n*-6, sum of omega-6 FA = C18:2*n-*6 + C20:2*n-*6 + 20:3*n-*6; ∑ AD, total atypical dienes = *c*9,*t*13-/*t*8,*c*13 + *t*8,*c*12-/*c*9,*t*12 + *t*9,*c*12 + *t*11,*c*15 + *c*9,*c*15; ∑ CLA, conjugated linoleic acid = *c*7,*t*9-/*c*9,*t*11- + *c*10,*t*12-18:2 + *t*9,*t*11-18:2; ∑ *t*-18:1, sum of *trans*-18 :1 isomers = *t*6-*t*8-18:1 + *t*9-18:1 + *t*10-18:1 + *t*11-18:1 + *t*12-18:1 + *t*13/*t*14-18:1 + *t*16-/*c*14-18:1; ∑ MUFA, sum of monounsaturated FA = ∑ *t*-18:1 + ∑ *t*-MUFA; ∑ *c*-MUFA, sum of *cis*- monounsaturated FA = *c*9-14:1 + *c*7-16:1 + *c*9-16:1 + *c*11-16:1 + *c*9-17:1 + *c*11-18:1 + *c*12-18:1 + *c*13-18:1 + *c*16-18:1 + *c*9-20:1 + *c*11-20:1; ∑ BCFA, sum of branched chain FA = *iso-*15:0 + *anteiso*-15:0 + *iso*-16:0 + *anteiso*-17:0 + *iso*-18:0; ∑ SFA, sum of saturated FA = C14:0 + C15:0 + C16:0 + C17:0 + C18:0 + C19:0 + 20:0.

Yearling-finished steers had greater (*P* < 0.05) subcutaneous proportions of *t*11,*c*15-18:2 (3-fold), one of the major non-conjugated 18:2 biohydrogenation products (*i.e.,* atypical dienes, AD) and lower (*P* < 0.05) proportions of *t*9,*c*12-18:2 (3.5-fold) compared to their contemporaries finished as weanlings (Table 2). These results could be related to differences in FA composition of the grower and finishing diets between the two production systems. The higher *t*11,*c*15-18:2 observed for the yearling-finished steers was likely derived from ALA obtained from pasture grazing while the elevated proportions of *t*9,*c*12-18:2 observed for the calf-finished steers may have been derived from higher LA proportions obtained from the high-grain finishing diet for these steers. The finding of increased *t*11,*c*15-18:2 in yearling steers demonstrates for the first time its persistence post-grazing; as such it may be a suitable long-term marker of forage consumption, and points towards its limited metabolism in subcutaneous adipose tissue. During rumen biohydrogenation, ALA yields conjugated linolenic acids, chiefly *c*9,*t*11,*c*15-18:3, which is in turn sequentially hydrogenated to yield AD isomers, chiefly *t*11,*c*15-18:2 through the activities of isomerase and reductase enzymes [27]. Overall, high proportions of a given FA in the tissues could be indicative of its limited metabolism or reflect slower rate of metabolism [28].

Neither total nor individual CLA isomers were affected by production system (*P* > 0.05; Table 2). With the exception of *t*12-18:1, production system had no influence (*P >* 0.05) on *t-*18:1 isomers. The subcutaneous proportions of *t*12-18:1 were greater (*P* < 0.05) in calf-finished steers than in yearling-finished steers but the reason for this is not immediately apparent. Calf *vs.* yearling finishing increased (*P* < 0.05) subcutaneous proportions of several individual *cis (c)-*MUFA isomers (*c*9-14:1, *c*11-16:1, *c*11-18:1, *c*12-18:1, *c*13-18:1, *c*9-20:1 and *c*11-20:1). These findings could be attributed to age-dependent differences in gene expression and catalytic activity of stearoyl-CoA desaturase [29,30]. The increase in *c*9-14:1, *c*11-16:1 and *c*13-18:1 reported for the weanling steers could be partly explained by the higher proportions of 14:0 (myristic acid) observed for these steers. Myristic acid is desatured to *c*9-14:1 by Δ9-desaturase, which is then elongated to *c*11-16:1 and *c*13-18:1, respectively. The proportions of *c*9-17:1 were greater (*P* < 0.05) for yearling steers than for weanling steers and the explanation for this is not immediately clear given that the proportions of C17:0, its derivative, were similar across production systems. The relative benefit or risk to human health of consuming the individual *c*-MUFA isomers remains to be elucidated, and thus recommendations to either enrich or deplete these isomers should be reserved until their effects are known.

Subcutaneous fat from yearling-finished steers as opposed to calf-finished steers had elevated (*P* < 0.05) proportions of total and individual (BCFA). The observed variability in BCFA proportions found between calf- and yearling-finished steers may also be partially attributed to the carryover effects of grower diets and backgrounding feeding regimes [31,32]. In the current study, yearling-finished steers as opposed to calf-finished steers entered the feedlot after a forage-based backgrounding phase. Overall, forage feeding has been reported to increase the percentage of BCFA in beef compared to concentrate feeding [6,33]. How grower diets and backgounding feeding regimes influence subsequent feedlot performance and FA composition of concentrate-finished steers merit further investigation. Improved understanding of mechanisms involved in enriching BCFA in meat would also be of interest given their potential to reduce cancer [34] and necrotizing enterocolitis [35] in humans.

Calf-finished steers had greater (*P* < 0.05) subcutaneous proportions of C14:0 (myristic acid) and smaller (*P* < 0.05) proportions of C15:0 compared to steers finished as yearlings. The observation that calf-finished steers had higher proportions of C14:0 than yearling-finished steers agrees with previous findings [36]. This may also in part be attributed to differences in total FA observed in the current study. Myristic acid is hypercholesterolemic and atherogenic [37] and thus, it is considered a less desirable component of the human diet. There were no differences (*P* > 0.05) in the proportions of total SFA and several major long-chain SFA (C16:0, C17:0, C18:0, C19:0 and C20:0) between calf- and yearling-finished steers.

### Effects of growth implants on fatty acid composition

Total FA content in adipose tissue in implanted and non-implanted steers was similar (*P* > 0.05; Table 2). Implanted steers had slightly higher (*P* < 0.05) subcutaneous proportions of total PUFA, total *n-*6 PUFA and LA, than steers without growth implants (Table 2). The subcutaneous proportions of other individual *n-*6 PUFA (C20:2*n-*6 and C20:3*n-*6) were similar (*P* > 0.05) across treatments. In support of the current findings, Ibrahim et al. [13] reported that fat from bull calves implanted with growth promotants had increased total PUFA and total *n-*6 PUFA. These findings could be partly related to the numerical differences in total FA content between implanted and non-implanted steers. Overall, the proportions of PUFA increases with decreasing fat content due to less dilution by *de novo* synthesised FA [38]. Growth implants had no effect on ALA, the only *n-*3 PUFA identified.

Neither total nor individual proportions of AD and CLA were influenced by growth implants (*P* > 0.05). For *t*-18:1, a pattern of isomers was found related to the implant strategy (Table 2). Implantation with growth promotants increased (*P* < 0.05) subcutaneous proportions of *t*-18:1 isomers with double bonds from carbon 6 to 10 compared to non-implantation. Growth promotants did not, however, influence (*P* < 0.05) total *t-*18:1. In agreement with the current study, implantation with estradiol benzoate and progesterone [11] or trenbolone acetate and estradiol [11,39] increased the proportions of some *t-*18:1 isomers (*t*6-*t*10) in the subcutaneous fat of concentrate-finished steers. These findings may relate to decreased dilution of *t-*18:1 isomers in subcutaneous fat by *de novo* synthesised FA. *Trans*-18:1 isomers other than *t*11-18:1 have been associated with unhealthy changes in blood lipid profiles in animal models [40] and are considered as undesirable components of the human diet. It is, however, not certain if the small differences (< 0.5%) in *t-*18:1 attributed to the use of growth promotants in the current study would be enough to impact human health.

Total and individual *c*-MUFA were not affected by growth implants except for *c*9-14:1 and *c*11-20:1. Steers implanted with growth promotants had low (*P* < 0.05) *c*9-14:1 and *c*11-20:1 compared to non-implanted steers. The observation that growth implants decreased the proportions of *c*9-14:1 agrees with earlier findings [39]. Proportions of total and individual BCFA were not affected by growth promotants except for *anteiso-*17:0 which was greater (*P* < 0.05) in steers implanted with growth promotants than steers without growth promotants (Table 2), but the differences were small (0.04%). Implantation had no effect on the proportions of total and several individual SFA (*P* > 0.05), however, the proportions of C18:0 tended to be greater (*P =* 0.09) in implanted than non-implanted steers. The finding that growth implants tended to increase the proportions of C18:0 may be related high proportions of PUFA reported for the implanted in the current study. Generally, high PUFA proportions inhibit Δ9 desaturase responsible for converting SFA to their respective MUFA [41].

## Conclusions

Yearling as opposed to a calf production system yielded lower subcutaneous proportions of individual *c*-MUFA and higher subcutaneous proportions of *t*11,*c*15-18:2, individual and total BCFA. Growth promotants increased subcutaneous proportions of total PUFA, total *n*-6 PUFA, LA and individual *t-*18:1 isomers (*t*6-*t*10), likely relating to reduced dilution by *de novo* synthesised FA. Overall, changes in subcutaneous FA composition of finished beef steers due to production systems and growth implants were limited with an average difference of 0.15% in FA composition which may not be sufficient to result in differences in human health.

## Competing interests

The authors declare that there are no competing interests in relation to this manuscript.

## Authors’ contributions

CM, TDT and MERD participated in the analyses of feed and subcutaneous fatty acids, statistical analysis of data and drafted the manuscript. JAB, JLA and VSB conceived the study, acquired funds, conducted the live animal study and participated in its design and write-up of the manuscript. All authors read and approved the final manuscript.
